# In silico work flow for scaffold hopping in Leishmania

**DOI:** 10.1186/1756-0500-7-802

**Published:** 2014-11-17

**Authors:** Barnali Waugh, Ambarnil Ghosh, Dhananjay Bhattacharyya, Nanda Ghoshal, Rahul Banerjee

**Affiliations:** Crystallography and Molecular Biology Division, Saha Institute of Nuclear Physics, Sector - 1, Block – AF, Bidhannagar, Kolkata, 700064 India; Computer Science Division, Saha Institute of Nuclear Physics, Sector-1, Block AF, Biddhannagar, Kolkata, 700064 India; Structural Biology and Bioinformatics Division, CSIR-Indian Institute of Chemical Biology, 4, Raja S.C. Mullick Road, Jadavpur, Kolkata, 700032 India

**Keywords:** Leishmaniasis, Drug targets, Pharamacophore, Scaffold hopping

## Abstract

**Background:**

Leishmaniasis,a broad spectrum of diseases caused by several sister species of protozoa belonging to family trypanosomatidae and genus leishmania , generally affects poorer sections of the populace in third world countries. With the emergence of strains resistant to traditional therapies and the high cost of second line drugs which generally have severe side effects, it becomes imperative to continue the search for alternative drugs to combat the disease. In this work, the leishmanial genomes and the human genome have been compared to identify proteins unique to the parasite and whose structures (or those of close homologues) are available in the Protein Data Bank. Subsequent to the prioritization of these proteins (based on their essentiality, virulence factor etc.), inhibitors have been identified for a subset of these prospective drug targets by means of an exhaustive literature survey. A set of three dimensional protein-ligand complexes have been assembled from the list of leishmanial drug targets by culling structures from the Protein Data Bank or by means of template based homology modeling followed by ligand docking with the GOLD software. Based on these complexes several structure based pharmacophores have been designed and used to search for alternative inhibitors in the ZINC database.

**Result:**

This process led to a list of prospective compounds which could serve as potential antileishmanials. These small molecules were also used to search the Drug Bank to identify prospective lead compounds already in use as approved drugs. Interestingly, paromomycin which is currently being used as an antileishmanial drug spontaneously appeared in the list, probably giving added confidence to the ‘scaffold hopping’ computational procedures adopted in this work.

**Conclusions:**

The report thus provides the basis to experimentally verify several lead compounds for their predicted antileishmanial activity and includes several useful data bases of prospective drug targets in leishmania, their inhibitors and protein – inhibitor three dimensional complexes.

**Electronic supplementary material:**

The online version of this article (doi:10.1186/1756-0500-7-802) contains supplementary material, which is available to authorized users.

## Background

Leishmaniasis, a broad spectrum of diseases, is caused by more than 20 sister species of protozoa belonging to the family Trypanosomatidae and genus *Leishmania*. These diseases are generally classified into three forms: visceral (VL), cutaneous (CL) and mucocutaneous (MCL), of which VL is lethal if left untreated, whereas CL, MCL generally self cure, though with the possibility of leaving permanent scars on the patient. The Indian subcontinent along with Sudan and Brazil account for the overwhelming majority of cases in VL, while the incidence of CL predominantly occurs in Afghanistan, Algeria, Brazil, Iran, Peru, Saudi Arabia and Syria [1]. Overall, about 10 million people are affected worldwide. The vector for this disease is the phlebotominae sand fly, which injects the parasites into the host in the course of a blood meal. The parasites thus exist in two forms: as mobile, flagellated promastigotes in the gut of the sandfly and non-motile, non-flagellated amastigotes which multiply within the phagolysosomal compartment of the macrophage in the mammalian host [2].

The first line of defense against the parasites traditionally continues to be generic pentavalent antimonials (sodium stibogluconate), especially in those regions where resistant strains have yet to appear. With the emergence of strains resistant to antimonials (especially in Bihar state of India) [3–5] second line drugs such as amphotericin-B (along with its liposomal formulations) and miltefosine are being extensively used [6]. However, both these drugs are more expensive than antimonials, toxic with reportedly severe side effects. Pentamidine and paromomycin are other drugs currently in use though their ready availability in endemic areas appears to be limited [7]. It is thus clear that there is an urgent need to search for and identify therapeutic alternatives to combat the disease.

With the availability of full genome sequences, search for drug targets in pathogenic organisms have been greatly facilitated. Comparative genomics allows the identification of genes unique to an organism, determination of parasitic genes absent in human and the evolutionary conservation of genes, probably reflecting upon their essentiality [8]. Gene conservation across pathogenic species also gives the added advantage that a single broad based antiparasitic targeting a conserved protein, could be used as a drug for several ailments. The genomes of five leishmanial species *L. major*, *L. infantum*, *L. donovani* , *L. mexicana* and *L. braziliensis* have been sequenced; with the first three consisting 36 chromosomes each, while *L. braziliensis* contains only 35. Notably, *L. braziliensis* has been assigned to a different subgenus *Leishmania* (*Viannia*) *sp.* and is thus somewhat distantly related to the others, which belong to the subgenus *Leishmania* (*Leishmania*) *sp.*. This reduction in chromosome number in *L. braziliensis* is due to a fusion event joining chromosome 20 and 34 (as numbered in *L. major*). Likewise, *L. mexicana* is two chromosomes less due to two fusions between four chromosomes (chromosome 8 and 29; chromosome 20 and 36). These genomes have approximately 8300 protein coding regions of which only about 40% can be ascribed a putative function [9–11]. In addition, the genomes of *T. brucei* (11 chromosomes) and *T. cruzi* are also available. Generally , the genomes of kinetoplastidae exhibit a high degree of synteny (conservation of gene order) in the organization of their genomes [12]. Comparison between the genomes of *T. brucei*, *T. cruzi* and *L. major* revealed a conserved core of approximately 6200 trypanosomatid genes and about 1000 ORFs [11] were notable for their presence in the genome of *L. major* alone. Further, upon comparing the genomes of leishmanial species, 5, 26 and 47 genes were identified to be exclusively and specifically present in the genomes of *L. major* , *L. infantum* and *L. braziliensis* respectively [13].

Leishmanial genomes consist of several novel metabolic pathways whose enzymes could serve as potential drug targets. Some of the distinctive features of these genomes include the presence of atypical protein kinases lacking the SH2, SH3, FN-III and immunoglobulin like domains which occur most frequently in humans [14, 15]. The cellular surface of leishmania consists of several unique glycoproteins which are essential for immune evasion and host – parasite interaction. The most abundant of these glycoproteins are attached to the surface of the plasma membrane via GPI (glucosylphosphatidyl inositol) anchors, which are essential for parasitic survival. Other novel pathways involve trypanothione metabolism, essential for cell growth and differentiation, which is replaced by glutathione in humans. The first enzyme in trypanothione synthesis is the enzyme ornithine decarboxylase targeted by the drug diflouromethyl ornithine, prescribed for human sleeping sickness. Enzymes of the glycolytic pathway, ergosterol synthesis in sterol metabolism and the purine salvage pathway also offer potential drug targets for therapeutic intervention [14]. Some of these pathways will be discussed in greater detail in the later sections of this paper.

Due to the exponential increase in genomic information, researchers are now confronted with a rapidly expanded list of gene products from which to select prospective targets. Several scoring schemes have been proposed which surveys the genome of a pathogenic organism and ranks genes according to their potential as drug targets [8, 16, 17]. Most schemes give a high weightage to the essentiality of the protein in the life cycle of the parasite, conservation of the target among different sister species and its corresponding absence in humans. Experimentally, either the lethality of gene deletion or insertion of transposons into the selected gene has been used to determine its essentiality. Non-essential genes could also be selected as targets, provided they play a vital role in the infective virulence of the pathogens. Other considerations includes the assayability of the protein, expression level during the life cycle of the organism possibly determined by microarray data and computational flux based analysis to gauge the effect of protein inhibition on the integrity of biochemical networks. In this connection, the TDR-target database is one of the most well cited amongst such databases [16]. This database consists of an exhaustive list of drug targets from the genomes of *L. major*, *T. brucei*, *M. leprae* and a host of other pathogens responsible for neglected tropical diseases. A useful feature of the database is its ability to prioritize a set of drug targets, where each criterion is assigned a weight and there is flexibility to change the weights associated with different factors (pathogenicity, essentiality, etc.) in the scoring scheme to extract a ‘custom-made’ list of targets relevant to the research interest of the user.

Several crystal structures of trypanosomal proteins, either individually or complexed with inhibitors are currently available in the Protein Data Bank, such as trypanothione reductase (*T. brucei),* trypanothione synthetase (*L. major*), pteridine reductase 1 (*L. major*), nucleoside hydrolase (*T. vivax*) and ATP dependent phosphofructokinase (*T. brucei*), which provide a detailed three dimensional structure of their active sites facilitating the design of specific inhibitors. In order to generate a library of prospective ligands which could have high affinities towards the active sites of targeted proteins, drug databases could be searched with structure based pharmacophores derived from protein ligand complexes. ‘Scaffold hopping’ or ‘chemotype switching’ [18–20], which involves identifying molecules with dissimilar backbone structures yet exhibiting very similar pharmacological properties, is one of the widely used techniques to generate compound libraries for eventual screening [21–25]. Lately, considerable success has been achieved in the application of structure based pharmacophores in the identification of lead compounds [21–25]. In the current work the human and the *L. major* genome have been compared to identify a set of proteins unique to the parasite. Crystal structures of these proteins or those of closely related homologues have been extracted from the Protein Data Bank (PDB) and the literature has been extensively surveyed to identify their specific high affinity inhibitors. Crystal structures of the protein (target) - inhibitor complexes have then been utilized to generate structure based pharmacophores. In case the crystal structures of the ligand bound targets were not available in the PDB, the inhibitors were computationally docked into the active sites of their receptors. Finally, the ZINC database and Drug Bank has been searched utilizing this set of pharmacophores to generate a set of compounds which could serve as a library in the search for prospective antileishmanial drugs.

## Methods

The annotated coding sequences (CDS) from the genomes of *L. major* (8316 CDS), *L. donovani* (8032 CDS), *L. mexicana* (8249 CDS), *L. braziliensis* (8056 CDS), *L. infantum* (8227 CDS), *T. brucei* (9962 CDS) and human (~41961 CDS) were downloaded from the NCBI genome database (http://www.ncbi.nlm.nih.gov/genome/; updated as on September 1, 2013). The standalone BLAST [26] was obtained from the NCBI ftp server (ftp://ftp.ncbi.nlm.nih.gov/blast/executables/blast+/LATEST/). Here BLAST refers to the BLASTp program of the NCBI standalone BLAST package which aligns protein amino acid sequences. To compare the annotated protein sequences between human and *L. major*(CDS’s) , the CDS’s from *L. major* were first fed in as a query (in FASTA format) whereas the human CDS’s were processed in the makeblastdb tool to form a BLAST database. This was followed by a second run of BLAST (with identical parameters) in which the human sequences were considered to be ‘query’ and the *L. major* proteins the database. Proteins which simultaneously passed identical filters in both the runs of BLAST were considered for the second step in the pipeline. A sole exception was made in the case of ATP dependent phosphofructokinase (PFK) in the first filter as the possibility of PFK being a drug target for trypanosomatids has been mentioned in the literature [27–29]. To compare *L. major* CDS’s with those of closest – related species, *L. major* proteins were fed in as a query and sequences from the other genome were made the database. All alignments with an E-value less than one were output from the program and default options were used for all other parameters. The software to analyze the BLAST outputs was developed locally in C/C++, Perl and the final results were displayed on Microsoft Excel sheets for further analysis.

In order to identify the unique metabolome in *L. major* with respect to its human host the ‘Comparative Analysis and Statistics’ option in the BioCyc Database (http://www.biocyc.org/) was used [30]. The metabolic pathways and enzymes associated with this unique set of metabolites from *L.major* were manually culled from the LeishCyc database (http://biocyc.org/LEISH/organism-summary?object=LEISH).

Template based homology modeling was performed using the MODELLER software both in the standalone mode and also implemented in Accelrys Discovery Studio 2.5. In addition, the GUI version of Modeller 9.11v (Easy Modeller 4.0) [31] was also used.

GOLD 5.2 (http://www.ccdc.cam.ac.uk/Solutions/GoldSuite/Pages/GOLD.aspx) was used to dock specific ligands onto the active site of their corresponding enzymes with the following parameters: population size 100, selection pressure 1.1, number of operations 100000(min) - 125000(max), islands 5, niche size 2, crossover frequency 95, mutation frequency 95, migration frequency 10 and search efficiency 100%. The program was run at least 10 times in order to confirm the best docking solution which was identified based on three criteria a) the CHEMPLP score b) rmsd between the docked solution and the initial placement of the ligand and c) visual survey and examination of the contacts between the docked ligand and protein. In case sufficient prior information was already available with regard to the position of the ligand in the active site of the protein (such as in Group B : See Section on The Protein – Ligand Complexes), the docking solution with the minimum rmsd (generally less than 1.0 Å) and whose CHEMPLP score was amongst the top three (comparable to the score derived from the original protein – ligand complex available in the PDB), was accepted as the most reasonable solution, subsequent to the visual inspection of the ligand bound active site. In cases where such information was either ambiguous or limited (Group C : See Section on The Protein – Ligand Complexes) the threshold on rmsd was relaxed to about 2.0 - 2.75 Å, the interactions of the ligand with active site were visually examined and the solutions grouped into sets with similar geometry with respect to the binding site. Amongst these sets, the pose with the maximum number of physically meaningful interactions and the best CHEMPLP score was accepted as the most favoured solution. All solutions which exhibited significant displacement of the ligand from the putative active site of the protein (>3.75 Å) were not considered. The decisions regarding both ligand flexibility and the flexibility of residues constituting the active site were decided on a case by case basis which will be discussed below (in the Results and Discussion section). Generally, in case the protein-ligand complex was available in the Protein Data Bank (PDB) and the leishmanial protein modeled utilizing the molecule from such a complex as a template, the ligand was transferred onto the modeled protein by utilizing the rotation matrix, translation vector derived from the superposition of the modeled protein to the template (Dali Server; http://ekhidna.biocenter.helsinki.fi/dali_lite/start) [32]. Most often in such instances both the ligand and the active site were held rigid whilst docking with GOLD. For ligands, whose complexes with the targeted protein (from *L. major*) or its close homologue were not available in the PDB, the ligand coordinates were derived from its closest related structure (as found as a complex in the PDB) by fourth atom fixing techniques and energy minimized by the semi-empirical quantum mechanical method in the program GAMESS [33, 34]. It goes without saying that the protein in such a complex would either be the target (*L. major*) or a close homologue from a sister trypanosomatid species. With the initial placement of ligand into the protein active site using the same techniques mentioned above, the newly added chemical groups to the parent compound (obtained from the PDB) were rendered flexible in the subsequent docking by GOLD, in addition to selected active site residues which could provide steric hindrance in the docking process. These residues were identified by visual examination of the initial docked position and examination of the list of contacts. In GAMESS the self-consistent field wave function with the semi-empirical basis set (AM1 model Hamiltonian) was used and the optimum tolerance of the energy minimization cycle was set to 1.0e-5. Where no information was available with regard to the association of the ligand with the target protein, blind docking was attempted subsequent to the placement of the ligand at the centroid of the putative active site of the enzyme.

LigandScout version 3.12 (build 20130912) was used to generate the structure based pharmacophores from the crystallographic and modeled/docked complexes, with manual monitoring of the entire process at every stage. To validate the pharmacophores, the ligand along with other active compounds (with relatively less IC_50_ values though with similar structures) were made the kernel of a database which also included decoys generated from the D.U.D.E. decoy generator (http://dude.docking.org/generate). The specific ligand, along with other actives and decoys were next submitted to OMEGA 2.5.1.4 [35] to generate two conformers per decoy and each of the active molecules from the docked/crystal structures. Thus, the database for each ligand (inclusive of the other actives and decoys) consisted of about 1800 molecular conformers in all. The library generating tool of LigandScout was then utilized to convert the database into a library (*.ldb format), prior to searching the library with the corresponding pharmacophore. Invariably, the specific ligand used to derive the structure based pharmacophore would be detected at the topmost rank. Every validation database was split into two, one consisting of actives and the other of decoys. Ligand screening option in LigandScout was then invoked to search both the databases with the specific pharmacophore as the query (with the advanced options, scoring function: ‘Pharmacophore - Fit’; Screening Mode: ‘Match all query features’; Retrieval Mode: ‘Get best matching conformation’).For every case, the maximum number of omitted features were varied to get optimal results and the ROC curve. Those pharmacophore queries, which gave screening results with area under the ROC curve less than 0.75 were not utilized in subsequent calculations. Finally, the ZINC database was searched by all the structure based pharmacophores derived from the protein ligand complexes.

## Results and discussion

### Comparative genomics – human versus Leishmania

**Figure 1:**
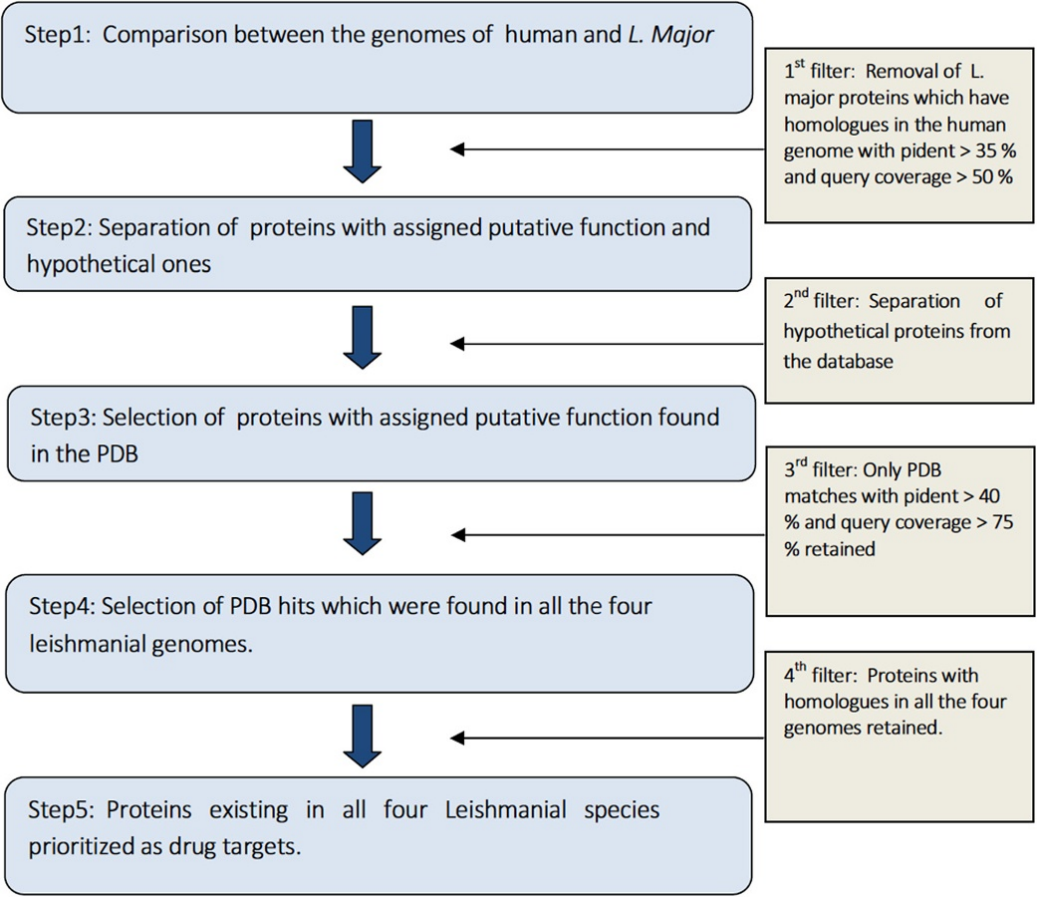
**Schematic representation of steps involved in the selection of drug targets from the genome of** ***L. major*** **(Additional file** **1** **)** ***.*** The separated set of hypothetical proteins were independently searched against the PDB (Additional file 2).

The whole set of annotated protein-coding genes from *L. major* genome (8316 CDS) was compared against the ones from human genome (41961 CDS) and those parasitic proteins (4991 among 8316 sequences) which could not align with any human gene, (first filter) with pident (percentage sequence identity) >35% and a simultaneous query coverage >50%, in two way reciprocal BLAST runs with *L. major* as query, human as database and vice versa (Materials & Methods), were selected for the next filter. Hypothetical sequences (4407 CDS) were removed from this list (second filter) and the Protein Databank (PDB) searched for each of the remaining sequences, which amounted to a total of 584 putative sequences. The PDB database was downloaded from the RCSB-PDB (http://www.rcsb.org/pdb/home/home.do) website and incorporated into BLAST using the methods given above (Materials and Methods). Only those genes were selected for subsequent analysis (a total 90 sequences) which recorded hits in the PDB with >40% sequence identity and a simultaneous query coverage >75% (third filter). Each gene from this set (consisting of *L. major* proteins alone) were then checked for BLAST hits (pident >40% & query coverage >75%) in the genomes of *L. donovani*, *L. mexicana*, *L. braziliensis*, *L. infantum* and also in the clusters of orthologous proteins in the Tritryp database (based on the OrthoMCL annotation [36]) . Upon merging both sets of data, only those (*L. major*) proteins with homologues/BLAST hits in all the five genomes were retained (fourth filter). The final set of genes consisted of a total of 86 polypeptide chains (Additional file 1). The schematic representation of the successive filters to arrive final list of drug targets is described in Figure 1. The separated list of hypothetical sequences (4407 CDS) were independently searched in the PDB and eighteen sequences scored hits satisfying the above criteria (given in Additional file 2). Upon the application of more stringent criteria (pident >25 and query coverage >33) in the first filter, 47 out of the original 86 genes satisfied the new threshold values, of which 13 genes were retained from the first thirty proteins of the original set (Additional file 1).

### Target prioritization

This list of parasitic proteins were then sorted according to a “*weighted union*” scoring scheme based on the following factors: i) essentiality as determined from experimental studies, ii) virulence factor iii) expression profile and iv) whether the natural substrate to the protein is a ligand unique to *Leishmania spp.* with respect to human. Information regarding essentiality and virulence were obtained by an extensive literature survey, in addition to consulting the TDR database. A list of metabolites unique to *Leishmania spp*. were identified by searching the leishmanial and human metabolome databases [37]. The metabolome of *L. major* was obtained from the Biocyc Database (http://biocyc.org/LEISH/class-tree?object=Compounds) (see Method) and the corresponding metabolome from human was available in the Human Metabolome database (HumanCyc; http://biocyc.org/HUMAN/organism-summary?object=HUMAN). Utilizing bioinformatics tools available in the *Biocyc,* the two metabolomes were compared and the ligands unique to *Leishmania spp.* were filtered out. From this comparison 129 metabolites were identified to be unique to the parasite. The full set of enzymes corresponding to these substrates were assembled into a blast database and run against the initial list of 86 genes. Only eight polypeptide chains from this list of 86 proteins were found to be associated with unique ligands. As has been mentioned previously, leishmania has two stages in its life cycle (amastigote and promastigote) and the information whether the gene was ‘constitutively expressed’ in both stages or in only one of them was obtained primarily from GeneDB [38], which provides a convenient platform to cull information with regard to leishmanial gene expression from the work of Leifsko *et al.* [39]. A score of 100 was awarded on the full satisfaction of any one of the criteria given above and thus the highest possible score obtained by any protein could be 400. Targets lacking experimental data with regard to essentiality were still given 50 in case strong arguments existed in favour of their being essential genes (e.g. phosphofructokinase in the glycolytic pathway) and 20 if the protein was found to be indispensable in a sister species. A score of 100 was assigned to the gene which was constitutively expressed in both stages and 50 if expressed in only one of them. The final list of prioritized proteins are given in (Additional file 1) and the first thirty proteins from this list (Table 1) are described in some detail in the context of unique metabolic pathways of leishmania parasites. The only available information with regard to the 18 hypothetical proteins (Additional file 2) was that they are constitutively expressed in both stages of the leishmanial life cycle [39] , and thus upon prioritization, these proteins (all with an identical score of 100) did not find their place amongst the first thirty, in the list of annotated proteins (given in Table 1).

**Table 1:** **Drug targets from the genome of** ***L. major***

No.	Target proteins	Metabolic pathway	Gene name (genedb id)	Unique ligand	Prioritization score
**1**	**Trypanothione reductase**	**Trypanothione metabolism**	**LmjF05.0350**	**Trypanothione**	**400**
**2**	**Pteridine reductase 1**	**Reductase**	**LmjF23.0270**	**Tetrahydropteroyltri-L-glutamate, 5-methyltetrahydropteroyltri-L-glutamate**	**400**
**3**	**Putative trypanothione synthetase**	**Trypanothione metabolism**	**LmjF27.1870**	**Trypanothione**	**350**
**4**	**UDP-galactopyranose mutase**	**Sugar metabolism**	**LmjF18.0200**	**β-D-ribopyranose**	**350**
**5**	**Putative udp-glc 4′-epimerase**	**Sugar Metabolism**	**LmjF33.2300**	**Absent**	**300**
6	Inhibitor of cysteine peptidase	Peptidase/protease	LmjF24.1770	Absent	220
7	Peptidase m20/m25/m40 family-like protein	Peptidase/protease	LmjF31.1890	Absent	220
8	Putative endoribonuclease L-PSP (pb5)	RNase	LmjF23.0200	Absent	220
9	Putative peptidase M20/M25/M40	Peptidase/protease	Lmjf33.1610	Absent	220
**10**	**Trypanothione-dependent glyoxalase I**	**Trypanothione metabolism**	**GLO1**	**S-lactoyl trypanothione**	**220**
**11**	**Putative deoxyuridine triphosphatase**	**Purine/pyrimidine metabolism**	**DUT**	**Absent**	**200**
12	Putative metacaspase protein	Peptidase/protease	LmjF35.1580	Absent	200
13a	Macrophage migration inhibitory factor-like protein	Host-parasite interaction, macrophage migration inhibitory factor	MIF1	Absent	200
13b	Macrophage migration inhibitory factor-like protein	Host-parasite interaction, macrophage migration inhibitory factor	MIF2	Absent	200
14	Putative calpain-like cysteine peptidase	Peptidase/protease	LmjF20.1185	Absent	200
15	Putative proteasome activator protein pa26	Peptidase/protease	LmjF35.0750	Absent	200
16a	Putative serine peptidase	Peptidase/protease	LmjF27.2630	Absent	200
16b	Putative serine peptidase	Peptidase/protease	LmjF29.1270	Absent	200
17	Putative NADP-dependent alcohol dehydrogenase	Glycolytic pathway/ gluconeogenesis/ glycerolipid metabolism	LmjF23.0360	1-alkyl-2acyl-phosphatidyl-inositol (an alcohol)	200
18	5-methyltetrahydropteroyltriglutamate-homocysteine S-methyltransferase	Amino acid metabolism	LmjF31.0010	5-methyltetrahydropteroyltri-L-glutamate	200
19	2,3-bisphosphoglycerate-independent phosphoglycerate mutase	Glycolytic pathway	PGAM	Absent	150
20	ADF/Cofilin	Cellular motility	LmjF29.0510	Absent	150
**21**	**ATP-dependent phosphofructokinase**	**Glycolytic pathway**	**LmjF29.2510**	**Absent**	**150**
22	Glucokinase	Glycolytic pathway	LmjF36.2320	Absent	150
23	Glycerol-3-phosphate dehydrogenase [NAD+],glycosomal	Glycerophospholipid metabolism	GPD	Absent	150
24	Nonspecific nucleoside hydrolase	Purine/pyrimidine metabolism	NH	Absent	150
25	Putative dipeptylcarboxypeptidase	Peptidase/protease	DCP	Absent	150
26	UDP-sugar pyrophosphorylase	Sugar metabolism	USP	Absent	150
27	Putative glutamate dehydrogenase	Amino acid metabolism	LmjF28.2910	Absent	120
28	Putative isocitrate dehydrogenase	TCA cycle	LmjF33.2550	Absent	120
29	Putative OMPDCase-OPRTase	Pyrimidine metabolism	LmjF16.0550	Absent	120
30	3-mercaptopyruvate sulfurtransferase	Amino acid metabolism	LmjF05.0970	Absent	120

Prioritized list of drug targets consisting of the first thirty proteins. Information regarding their metabolic pathway, genebank ID and association with a ligand unique to leishmania w.r.t human are also included in the table. Proteins given in bold were subsequently used for pharmacophore calculations.

As expected, prominent amongst the list of prioritized proteins (Table 1) are three enzymes associated with trypanothione i)trypanothione reductase (TR: 1), ii)putative trypanothione synthetase (TS: 3) and iii)trypanothione - dependent glyoxalase I (GLO1: 10). The trypanothione system in leishmania which replaces the ubiquitous glutathione system present in humans, enables the parasite to survive the high oxidative stress found in the host immune system and the presence of toxic heavy metals [40]. Both trypanothione synthetase (which synthesizes trypanothione from glutathione and spermidine) and trypanothione reductase (which keeps it in its reduced form in the presence of NADPH) are attractive drug targets [41], as this system is the only pathway involved in the crucial regulation of oxidative stress in the parasites. Reduced trypanothione in turn causes the reduction of tryparedoxin which then transfers electrons to the recycling enzyme tryparedoxin peroxidase [40]. Although TR and human glutathione reductase(GR) exhibits 35% sequence identity and shares many physicochemical properties , yet their corresponding active sites are different due to their diverse substrate specificities [42]; TR binding only to the oxidized forms of positively charged glutathionyl – polyamine conjugates whereas human GR associates only with negatively charged glutathione [43]. The difference in specificity is primarily due to the presence of five amino acid residues in the TR active site, which confers enhanced hydrophobicity, negative charge and wider access to its binding pocket relative to human GR [43]. Several inhibitors specifically designed for TR have yet to be entirely successful as drugs, probably due to the wide active site of the enzyme which poses obstacles for structure-based drug design, coupled to the pharmacokinetic properties of the inhibitors [43]. In addition, trypanothione is also implicated in the Glyoxalase I, II systems in the parasite (again replacing glutathione in humans) which is responsible for the removal of toxic and mutagenic methylglyoxal formed as a byproduct of glycolysis. The crystal structure of leishmanial glyoxalase I (GLO I) reveals differences with respect to the corresponding human enzyme in its active site architecture [44], which includes increased negative charge and hydrophobicity along with the truncation of a loop which could be involved in the catalytic activity of the human enzyme.

The surface glycocalyx of *Leishmania spp.* consists of several unique sugars and glycoconjugates which mediate host – parasite interaction and virulence. A significant fraction of these glycoconjugates consists of lipophosphoglycans (LPG) implicated in the adhesion of leishmania to the host cell and glycoinositolphospholipids (GIPLs) involved in pathogenesis [45]. Both LPGs and GIPLs, have β-galactofuranose (β-Gal*f*) as one of their main constituents, an unusual sugar not found in vertebrates. Three proteins in (Table 1), UDP-galactopyranose mutase (UGM: 4), putative UDP-Glc 4′-epimerase (galE: 5), UDP-sugar pyrophosphorylase (USP: 26) are constituents of biochemical pathways, either directly or indirectly responsible for the synthesis of β–Gal*f*. β – Gal*f* is synthesized from its precursor UDP-galactose (UDP–Gal) by the enzyme UDP-galactopyranose mutase (UGM), inhibition of which is known to regulate parasitic virulence and hence is an attractive target [46]. The cellular pool of UDP-Gal is contributed by the Isselbacher and Leloir pathways [45, 46]. In the Leloir pathway, UDP–Gal is synthesized from galactose - 1 - phosphate by UDP - sugar pyrophosphorylase (USP), whereas in the Isselbacher pathway galactose–1–phosphate is converted to UDP-Gal and glucose–1-phosphate by galactose–1–phosphate uridyltransferase. Within this pathway, the reversible and bidirectional enzyme UDP-Glc 4′-epimerase (GalE) can convert UDP – Gal to UDP – Glucose and vice versa. Despite low sequence identity of about 33% between human and parasitic GalE , high resolution crystal structures of both proteins reveal a common overall topology and similar protein-ligand interactions at the active site [47]. GalE holds great promise as a drug target in *T. brucei*.

Next, a set of five proteins implicated in purine/pyrimidine metabolism occupied fairly prominent positions in Table 1: putative deoxyuridine triphosphatase nucleotidohydrolase (dUTPase: 11), nonspecific nucleoside hydrolase (NNH: 24), and putative OMPDCase – OPRTase (OMPDC-OPRT: 29). Unlike their human and other mammalian hosts leishmania lack the molecular machinery to synthesize purine nucleotides *de novo* and is thus dependent on a purine salvage pathway [48]. Extensive genetic studies on the purine salvage pathway show it to be highly complex with several redundant links. For example, mutant strains individually lacking one of the key enzymes of the pathway: adenine phosphoribosyl transferase (APRT), hypoxanthine – guanine phosphoribosyltransferase (HGPRT), adenosine kinase (AK) and xanthine phosphoribosyl transferase (XPRT) were all found to be viable [48]. However, the phenotypic characterization of the double *∆hgprt/∆xprt* mutant indicated that purine salvage from extracellular sources is primarily funneled through XPRT, HGPRT with AK and APRT being by and large superfluous [49]. Thus, the central role played by these two enzymes (HGPRT & XPRT) confers functional importance to downstream molecules which distributes their products into adenylate and guanylate nucleotides. Adenylosuccinate synthetase (ADSS) and adenylosuccinate lyase (ASL) are two such enzymes which sequentially convert IMP to AMP, the former catalyzing the GTP dependent formation of adenylosuccinate from IMP and aspartic acid while the latter removes a fumarate from adenylosuccinate formed by ADSS. Knock out mutants of ASL shows highly attenuated infectivity of the parasites [49].

Null mutants of purine transporters LdNT1, LdNT2 (*∆ldnt1*, ∆*ldnt2* and *∆ldnt1* /∆*ldnt2*) do not appear to interfere with parasitic growth based on natural purine sources (with the exception of xanthosine) [48]. Subsequently, the enzyme nonspecific nucleoside hydrolase (NNH) was identified to perform the non-specific conversion of purine nucleosides to nucleobases, which can then be transported by other transporters LdNT3 and LdNT4 [48]. In *Leishmania spp.* NNH hydrolyzes the N-glycosidic bond of both purine and pyrimidine nucleosides to yield ribose and other bases. Upon intake and conversion, adenine bases are irreversibly deaminated to hypoxanthine by the enzyme adenine aminohydrolase (AAH). However, despite its unique presence in the parasite (w.r.t mammals and humans), the enzyme was found to be non – essential as demonstrated by the viability of ∆*aah* knockouts [50].

Both humans and leishmania are capable of the de novo synthesis of pyrimidines though there exists considerable discrepancy in the organization of their corresponding enzymes into multifunctional polypeptides, cellular localization and allosteric regulators [51]. This is especially true of the last two enzymes in the pyrimidine synthesis pathway, orotate phosphoribosyltransferase (OPRT) and orotidine monophosphate decarboxylase (OMPDC), which are fused into one bifunctional protein, in both human and parasite. However the order of the polypeptide chains are reversed in both cases [51]. As putative OMPDCase - OPRTase (OMPDC - OPRT) is active in the final step of pyrimidine biosynthesis , its inhibition is expected to be lethal for the parasite. Finally, putative deoxyuridine triphosphatase nucleotidohydrolase (dUTPase) hydrolyzes dUTP to dUMP and pyrophosphate leading to the maintenance of the dTTP:dUTP ratio in the cell ensuring precision in DNA replication [52].

Another unique feature of protozoa belonging generally to kinetoplastids is the compartmentalisation of the first seven glycolytic enzymes (and therefore glycolysis) into organelles called glycosomes, in contrast to other organisms where glycolysis generally occurs in the cytosol. This feature is essential for the regulation of glycolysis in the parasite and also to effectively switch over to anaerobic forms of respiration [53]. Three such glycolytic enzymes 2,3 - bisphosphoglycerate – independent phosphoglycerate mutase (PGM: 19), ATP – dependent phosphofructokinase (PFK: 21) and glycerol - 3 - phosphate dehydrogenase (23), including two other enzymes either upstream or downstream of the glycolytic pathway, putative NADP-dependent alcohol dehydrogenase (17) and glucokinase (22) appeared in Table 1. Since glycolysis is the only known source for ATP in leishmania these enzymes offer attractive targets, especially phosphoglycerate mutase (i-PGM) which is distinct in terms of structure, catalytic mechanism and whose reduced expression was also found to be lethal for cultured *T. brucei*. However, despite intense effort on some of these validated targets effective pharmaceutical interventions have yet to emerge.

Traditionally, drugs inhibiting folate metabolism, specifically dihydrofolate reductase (DHFR) and thymidylate synthase (TS) have been successful as antibacterials. DHFR maintains the THF (N^5^, N^10^-methylene tetrahydrofolate) pool in the cell by the NADPH dependent reduction of dihydrofolate (DHF), which in turn is utilized by TS to catalyze the conversion of dUMP to dTMP. Lack of dTMP curtails DNA replication leading to cell death [54]. In leishmania both these enzymes are conjoined into a bifunctional enzyme DHFR-TS which is the primary source of reduced folate and also the lone source of thymidylate in the parasite [55]. However, inhibition of this enzyme is ineffective in promoting lethality due to the presence of another short chain non – specific dehydrogenase/reductase pteridine reductase 1 (PTR 1: 2), which acts both as a modulator and bypass for inhibitors targeting DHFR – TS. PTR 1 is responsible for the essential salvage of unconjugated pterins (such as biopterins) as it catalyzes the NADPH dependent two step reduction of oxidized pterins to their active tetrahydro forms. Deletion mutants of PTR-1 alone were non – viable and hypersensitive to the drug methotrexate (MTX) and had to be simultaneously inhibited [54], in case DHFR-TS was being targeted.

Among the first 30 prioritized targets a group of peptidases: peptidase m20/m25/m40 (7, 9), putative calpain – like cysteine peptidase (14), putative serine peptidase (16a, 16b), putative proteosome activator protein pa26 (15), putative dipeptidylcarboxypeptidase (25) and a putative metacaspase protein (12) were found in Table 1. A wide range of proteases spanning most of the major classes have been identified in the leishmanial genomes, with *L. braziliensis* alone having at least 44 cysteine , 23 serine and 97 metalloproteases [56]. Of these, cysteine proteases (CP) have been confirmed as virulence factors playing a major role in mediating host – parasite interactions; with parasites (*L.tropica*) treated with CP inhibitors exhibiting reduced viability, growth and pathogenicity. Metalloproteinases have also been known to be expressed on the surface of *Leishmania spp*., protecting the pathogen from the defensive action of host enzymes and the phagolysozome of macrophages [56]. In addition, a CP inhibitor; inhibitor of cysteine peptidase (6) also appeared at a prominent position in Table 1 by virtue of its being a virulence factor and a probably protecting the parasite from the hydrolytic environment of the sandfly gut or the internal environment of host macrophages.

The rest consisted of a miscellaneous collection of enzymes such as putative endoribonuclease L-PSP (8), involved in mRNA salvage and protein synthesis [57]; macrophage migration inhibitory factor like protein (13a,13b), implicated in the evasion of innate host immunity by arresting the apoptosis of infected macrophages [57]; 5-methyl-tetrahydropteroyltriglutamate-homocysteine S-methyltransferase (18), which plays important role in the synthesis of cysteine/methionine and also their interconversion [58], putative glutamate dehydrogenase (27), 3-mercaptopyruvate sulfurtransferase (30) involved in amino acid metabolism and ADF – cofilin(20)for cellular growth and motility [57].

### The protein – ligand complexes

An exhaustive literature survey was conducted to identify inhibitors for the first thirty proteins from Table 1. Inhibitors with experimentally determined IC_50_ or *K*_*i*_ values were found in the literature for only 8 out of the 30 proteins. A total of 27 inhibitors (Table 2, Additional file 3) were shortlisted for the above mentioned eight target proteins by selecting those ligands with the lowest IC_50_ values from a given family of compounds (that is a class of compounds with a conserved backbone/kernel and diverse peripheral substituents). Thus a total of 32 protein-ligand complexes (27 from *L. major* and 5 from *T. brucei*, iTb4-8) were divided into three sets (Table 3; Group A, B & C):

**Table 2:** **Drug target inhibitors**

No.	PROTEIN	Inhibitor name	IC50 /Ki
i1	Pteridine Reductase 1	methotrexate (nature structural biology, volume 8, number 6, june 2001)	1.1 μM
i2	Pteridine Reductase 1	Trimethoprim (Experimental Parasitology 87, 157–169 (1997))	12 μM
i3	Pteridine Reductase 1	10 – propargyl-5,8-dideazafolic acid (J. Mol. Biol. (2005) 352, 105–116)	> 10 μM
i4	Trypanothione Reductase	methyl [(4S) - 6 - bromo - 2 - methyl - 4 - phenylquinazolin - 3(4H)-YL] acetate (J. Med. Chem. 2011, 54, 6514–6530)	6.8 μM
i5	Trypanothione Reductase	(4 s) - 3 - benzyl - 6 - chloro - 2 - methyl - 4 - phenyl - 3,4 – dihydroquinazoline (J. Med. Chem. 2011, 54, 6514–6530)	0.93 μM
i6	Trypanothione Reductase	n - {2 - [(4 s) - 6 - chloro - 2 - methyl - 4 - phenylquinazolin - 3(4 h) - yl] ethyl}furan - 2 – carboxamide (J. Med. Chem. 2011, 54, 6514–6530)	0.86 μM
i7	Trypanothione Reductase	(4 s) - 6 - chloro - 3 - {2 - [4 - (furan - 2 - ylcarbonyl)piperazin - 1 - yl]ethyl} -2 - methyl - 4 - phenyl - 3,4 –dihydroquinazoline (J. Med. Chem. 2011, 54, 6514–6530)	0.42 μM
i8	Trypanothione Reductase	3 - [(4 s) - 6 - chloro - 2 - methyl - 4 - (4 - methylphenyl) quinazolin - 3(4 h) -yl] - n,n - dimethylpropan - 1 – amine (J. Med. Chem. 2011, 54, 6514–6530)	0.23 μM
i9	Trypanothione Reductase	a C6 - substituted and C8 - substituted 3,4 - dihydroquinazoline analogue (J. Med. Chem. 2011, 54, 6514–6530)	0.35 μM
i10	Pteridine Reductase 1	a quinazoline derivative (Experimental Parasitology 1997, 87, 157–169 )	0.4 μM
i11	Pteridine Reductase 1	a 2,4-Diaminopyrimidine derivative (Experimental Parasitology 1997 ,87, 157–169 )	0.4 μM
iTb4 to iTb8	Trypanothione Reductase crystal complex from T. brucei	Above inhibitor i1 to i5. (J. Med. Chem. 2011, 54, 6514–6530)	Same as above
i12	ATP dependent Phospho Fructo Kinase	a N, N0 - substituted - 1-amino - 2, 5 - anhydro - 1 - deoxy - 1 - D -mannonamide derivatives (Bioorg. Med. Chem. 2008, 16 , 5050–5061)	49 μM
i13	ATP dependent Phospho Fructo Kinase	2, 5 - anhydro - 1 - deoxy - 1 - (3, 4 - dichlorobenzylamino) - D – mannitol (Bioorg. Med. Chem. 2008, 16 , 5050–5061)	0.4 μM
i14	ATP dependent Phospho Fructo Kinase	2, 5 - Anhydro - 1 - deoxy - 1 - (3, 4 - dichlorobenzylamino) - D - 3, 4 - dichlorobenzylmannonamide (Bioorg. Med. Chem. 2008, 16 , 5050–5061)	23 μM
i15	Deoxyuridine Triphosphate Nucleotidohydrolase	a 5′ - tert - butyldiphenylsilyloxy derivative (J. Med. Chem. 2005, 48, 5942–5954)	3.0 μM
i16	Deoxyuridine Triphosphate Nucleotidohydrolase	a 5′ - Ph3CNH derivative (J. Med. Chem. 2005, 48, 5942–5954)	NA; substrate analog
i17	Deoxyuridine Triphosphate Nucleotidohydrolase	5′ - tritylamino - 3′ - fluoro - 2′, 3′, 5′ – trideoxyuridine (J. Med. Chem. 2005, 48, 5942–5954)	3.6 μM
i18	Deoxyuridine Triphosphate Nucleotidohydrolase	5′ - O - triphenylsilyl - 2′, 3′ - didehydro - 2′, 3′ – dideoxyuridine (J. Med. Chem. 2005, 48, 5942–5954)	12 μM
i19	Nonspecific Nucleoside Hydrolase	immucillin A (J. Biol. Chem. 1999,274(30, 21114–21120)	15 nM
i20	Nonspecific Nucleoside Hydrolase	immucilin ACAP (J. Biol. Chem.1999, 274(30), 21114–21120)	6.5 nM
i21	Nonspecific Nucleoside Hydrolase	N - (9 - deaza - adenin - 9 - yl) methyl - 1, 4 - dideoxy - 1, 4 - imino - D - ribitol (Antimicrobial Agents and Chemotherapy, 2010, 1900–1908)	0.49 μM
i22	Nonspecific Nucleoside Hydrolase	immucillin-H (1, 4 - dideoxy - 4 - aza - 1 - (s) - (9 - deazahypoxanthin - 9 - yl) - d - ribitol) (Biochem Biophys. Acta.2009, 1794, 953–960)	K_i_ = 6.2 nM
i23	Nonspecific Nucleoside Hydrolase	7 - (((2R, 3R, 4S) - 3, 4 - dihydroxy - 2 - (hydroxymethyl) pyrrolidin - 1 - yl) methyl) - 3H - pyrrolo [3, 2-d] pyrimidin - 4 (5H) - one (Biochem Biophys. Acta. 2009, 1794 , 953–960)	Ki = 4.4 nM
i24	Nonspecific Nucleoside Hydrolase	(2R, 3R, 4S) - 2 - (hydroxymethyl) - 1 - (quinolin - 8 - ylmethyl) pyrrolidine - 3, 4 – diol (Biochem Biophys. Acta. 2009, 1794, 953–960)	K_i_ = 10.8 μM
i25	Trypanothione Synthetase	1-[3-(3-fluorophenyl)indazol-1-yl]-3,3-dimethylbutan-2-one (J.Biol.Chem., 2009, 284( 52), pp. 36137–36145)	0.095 μM
i26	Putative-UDP-glc-4′-epimerase	ebselen (Bioorg. & Med. Chem. Lett. 2006, 16 , 5744–5747)	0.62 μM
i27	GLO1	S-4-bromobenzylglutathionylspermidine (Molecular Microbiology,2006, 59, 1239–1248)	Ki = 0.54 μM

Inhibitors identified for eight of the proteins from Table 1 along with their respective IC_50_’s or Ki values with respect to their corresponding target proteins. The inhibitors will be referred to by the numbers assigned in the first column.

**Table 3:** **Protein – inhibitor groups**

Groups	Status considered	Proteins
**A**	Protein-inhibitor complex structure available	PTR I
	Modelling not required for protein	
	Inhibitor’s position and coordinates known	
	Docking not necessary	
**B**	Protein-inhibitor complexes not available	TR (from *L. major & T. brucei*) ATP dependant PFK dUTPase, NNH
	Modelling required for protein from close homolog or structure available	
	Inhibitor pose & coordinates taken from known homologous complex	
	Docking performed with positioned ligand	
**C**	Protein-inhibitor complex not available	TS, GalE, GLO1
	Modelling required for protein from close homolog	
	Inhibitors are drawn ab initio and energy minimized	
	Blind docking performed based on the putative active site found in the literature

(I) In the first set (Group A), the crystal structures of the ligand bound protein complexes were readily available in the protein data bank and were utilized directly for computing the structure based pharmacophores (Table 3, Additional file 3; inhibitors i1,i2 and i3). Henceforth the inhibitors will be referred to by the number enumerated in Table 2.
(II) In Group B, the crystal structures of the ligand-protein complexes were available, with the protein either being the actual targeted molecule (from *L. major*) or a closely related homologue, with sequence identity exceeding 60% with respect to the corresponding protein from *L. major*. In the latter cases the homologous protein present in the PDB was used as a template to model the parasitic protein. Likewise, the specific ligand used to form the complex could either be the original small molecule found in the PDB file; or the ligand coordinates from the crystal structure were used as a template to add peripheral chemical groups. Specific ligands were docked onto the corresponding parasitic proteins using the GOLD software. In addition, the original protein-ligand complexes present in the PDB (from other trypanosomes) were also included in the subsequent calculations (Table 3, Additional file 3; inhibitors i4 – i9, iTb4 – iTb8 & i10 – i24). The ability of the docking protocol as implemented in the GOLD software to independently locate the ligand position as found in the crystal structure was verified for all the complexes used in this study.
(III) In Group C no information regarding both the proteins and their specific ligands were available in the PDB, though crystal structures exceeding a sequence identity of 50% with respect to the target were present in the database, which were used as templates to obtain the three dimensional models of the leishmanial proteins. The ligand coordinates were either obtained from the PubChem database (NCBI) or constructed *ab initio* (Material & Methods) by fourth atom fixing techniques. Blind docking (by GOLD) was used to position the inhibitor onto the putative active site (as obtained from the literature) of the enzyme (Table 3, Additional file 3; inhibitors i25, i26 & i27).

Thus 32 structure based pharmacophores were computed from their corresponding protein - ligand complexes, of which 3 complexes belonged to Group A, 21to Group B and 3 to Group C. In addition, 5 complexes (for inhibitor no. iTb4 to iTb8) whose proteins belonged to other trypanosomatids were also included in the calculation, by virtue of their being templates for the leishmanial proteins in Group B.

**Figure 2:**
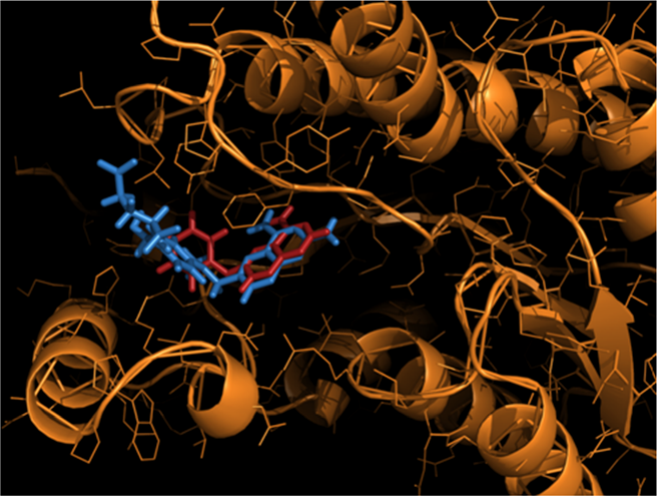
**Active site of Pteridine Reductase 1 (PTR1) complexed with methotrexate (i1 - blue) as found in the crystal structure IE7W and the docked quinazoline derivative (i10 – red).**

Complexes in Group A (Table 3, Additional file 3) consists of pteridine reductase 1 (PTR 1) bound to inhibitors methotrexate (1E7W; inhibitor no. i1), trimethoprim (2BFM; inhibitor no. i2) and 10 – propargyl-5, 8-dideazafolic acid (2BFA; inhibitor no. i3). Crystal structures of these three complexes include the cofactor NADPH. PTR 1 is a homotetramer, with individual subunits displaying the double Rossmann Fold composed of a central 7 - stranded parallel β-sheet with three α-helices on either side (Figure 2) [54]. All three structures exhibit high structural conservation, with the active site being an elongated L-shaped cleft constituted by the C terminal section of the strands β_1_ - β_6_, parts of the helices α_1_, α_4_ and a loop interconnecting a strand (β_6_) and a helix (α_6_) [54]. Two more complexes involving PTR1 with a quinazoline derivative (inhibitor no. i10) and a 2, 4-diaminopyrimidine derivative (inhibitor no. i11) were included in Group B. The methotrexate structure from the PDB file 1E7W was used as a template to build the quinazoline derivative (inhibitor no. i10) and the best solution with a CHEMPLP score of 49.44 was finally selected out of several GOLD runs (See Methods). The rmsd between the atoms common to methotrexate (pteridine ring or its derivative) as located in the PTR1 active site and the docked inhibitor-i10 was 0.77 Å. Protein ligand contacts involving residues Ser 111, Phe 113, Asp 181, Leu 188, Tyr 194, Leu 226 , Leu 229, Asp 232 and Met 233 were common both for methotrexate and Inhibitor i10 (Additional file 4). Notably, contacts between the pteridine ring and Phe 113, Ser 111 were prominent in both cases. Additional contacts were observed in methotrexate with respect to the inhibitor due to the more elongated character of the molecule, extending from the pteridine ring (Additional file 4; Figure 2). A similar procedure adopted for inhibitor-i11 based on trimethoprim (PDB code 2BFM) as a template gave a corresponding CHEMPLP score of 79.80. Although the orientations for both ligands were fairly similar, a translational shift in the position of inhibitor i11 was due to the substitution of -CH_2_Ph in place of -H in the pyrimidine ring of trimethoprim. The common residual contacts for both ligands were Phe 113, Asp 181, Leu 188, Tyr 194, Leu 226, Leu 229, and His 241 (Additional file 4). All the four ligands also maintained atomic contacts with NADPH.

**Figure 3:**
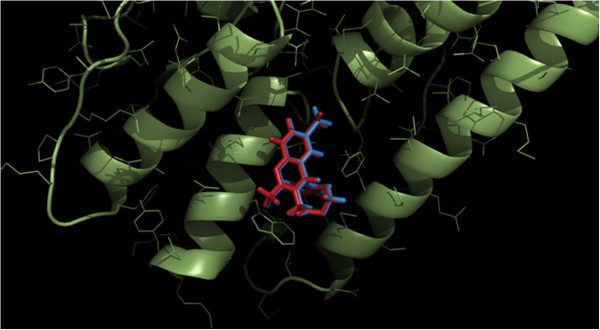
**Active site of the modeled Trypanothione Reductase (TR) from** ***L. major*** **complexed with ligand i4 (red) along with iTb4 (blue) placed utilizing the superposition matrices and vectors obtained from superposing the protein model onto its template (2WP5).**

Other protein targets in Group B apart from PTR – 1 were TR, PFK, dUTPase and NNH. Five complexes of TR from *T. brucei* were included (inhibitor numbers iTb4 – iTb8; See Table 2, Additional file 4) directly from the crystal structures with PDB codes 2WP5, 2WP6, 2WPE, 2WPC and 2WPF [59]. TR from *T. brucei* has a sequence identity of 66.5% with respect to the corresponding protein from *L. major* and was used as a template to model the parasitic protein. TR (*T. brucei*) is a homodimer with each subunit consisting of three domains, the inhibitor binding cleft being formed by a congregation of α helices in domain I [60]. The binding site exhibits conformational flexibity indicating an induced fit of the ligand to the binding pocket. Thus for each ligand (i4 – i8) the leishmanial protein was repeatedly modeled from its original complex (2WP5, 2WP6 etc. as the case maybe). The ligands were initially placed in the active site of the modeled proteins based on the rotation matrices and translation vectors obtained upon superposing the Cα coordinates (Dali server; http://ekhidna.biocenter.helsinki.fi/dali_lite/start) of the template onto its associated model. Subsequent GOLD runs gave high CHEMPLP scores greater than 60.0 for all the five complexes and rmsd’s ranging from 0.2 – 0.8 Å between the final docked structure and the initial position of the ligand in the modeled protein (Figure 3).

The active sites for all the five ligands (from 2WP5 – 2WPF) were completely conserved in both *L . major* and *T. brucei* , with residues Leu 17, Glu 18, Trp 21, Tyr 110, Met 113, Phe 114 making atomic contacts with all the ligands (with the exception of inhibitor i9 in *L. major*), in both the enzymes (Additional file 4). In addition, Ser 14, Leu 17, Gly 49, Leu 120 and Ile 339 were found in the vicinity of the ligands, occasionally in some of the complexes. i9 (Table 2, Additional file 4) was obtained from (4 s)-3-benzyl-6-chloro-2-methyl-4-phenyl-3,4-dihydroquinazoline (inhibitor i8:2WP6) and the final docked position (allowing for flexibility in residues Glu 18, Trp 21 and Met 113 in the active site) had a rmsd of 0.66 Å (with respect to common atoms of 3,4-dihydro-quinazoline analogues) and a score of 34.69. The same set of core residues (Leu 17, Glu 18 etc.) including Ser 14 and Leu 120 also formed the active site for inhibitor i9 (Additional file 4).

The active site for fructose-6-phosphate (F6P) of the trypanosomatid ATP dependent PFK from *T. brucei* exhibits significant structural differences compared to its human counterpart and is located at the interface of two subunits in the homotetramer. Each subunit is composed of three domains (A, B, C) with the ATP binding site (between domains B and C) lying adjacent to the F6P site [61]. The complete tetramer of PFK from *L. major* was modeled based on the homologous protein from *T. brucei* (3F5M) with respect to which it shares a 71% sequence identity and ATP (along with the Mg^2+^ ) were docked/placed in the model, prior to placement of the substrate. The crystal structure of PFK from *T. brucei* in 3F5M is in complex with ATP and does not contain the substrate F6P, whose coordinates were extracted from 1MTO which consists of PFK from *B. stearothermophilus* in complex with F6P [61] and docked onto the corresponding active site in leishmanial PFK, subsequent to initial positioning of the molecule, following similar methods given above. Beginning with the coordinates of the furan ring of F6P, three other inhibitors were built by making appropriate substitutions : a N,N0-substituted-1-amino-2, 5-anhydro-1-deoxy-1- D-mannonamide derivative (inhibitor no. i12); 2,5-anhydro-1-deoxy-1-(3,4-dichlorobenzylamino) -D-mannitol (inhibitor no. i13) and 2,5-Anhydro-1-deoxy-1-(3,4-dichlorobenzylamino)-D-3,4-dichlorobenzylmannonamide (i14). Subsequent docking with GOLD on a rigid active site gave CHEMPLP scores 70.35, 67.80 and 46.81 for the inhibitors i12, i13 and i14 respectively. Repeated attempts to dock the ligands on a flexible site did not yield physically meaningful results. The only common feature between the inhibitors – i12, i13 and i14 was the furan ring (from F6P) and considerable variability in the remaining features tended to shift the ligands from their initial position depending on the length and chemical character of the substituents on either side of the furan ring. Consequently with a few exceptions, the constellation of residues constituting their binding pockets were significantly different (Additional file 4).

The crystal structure of dUTPase from *L. major*, is a dimeric all α protein (in contrast to its trimeric all β human homologue) was obtained from the PDB (2CJE) [62]. The active site is located in the vicinity of the interface between the rigid and mobile domains which constitute each subunit [62]. In addition, the site on one subunit is also constituted by a loop contributed by the other monomer. Crystal structures of the closed and open forms of the enzyme from *T. cruzi* revealed a significant movement of the mobile domain and rearrangement of the secondary structural elements [62]. The closed ligand bound form of dUTPase from 2CJE was used to model complexes with three other inhibitors. Since the enzyme sits in a special position in the crystal structure, the entire dimer of dUTPase was generated prior to the placement of the other ligands. The bound substrate analogue DUPNHP (2′-deoxyuridine 5′-alpha,beta-imido-diphosphate from 2CJE) was used to design the three inhibitors : a 5′-tert-butyldiphenylsilyloxy derivative (i15), a 2′-deoxyuridine 5′-alpha,beta-imido-diphosphate (i16), 5′-tritylamino-3′-fluoro-2′,3′,5′-trideoxyuridine (i17) and 5′-O-triphenylsilyl-2′,3′-didehydro-2′,3′-dideoxyuridine (i18). CHEMPLP scores for all the four inhibitors ranged from 90–100 and the rmsd’s between the starting and final docked position ranged from 0.5 – 1.2 Å. Based on the visual examination of the initial ligand position in the active site of the enzyme and survey of the ligand – protein atomic contacts, selected residues were rendered flexible in the docking process which could provide steric hindrance to the optimal orientation of the ligand or adopt alternative conformations in the binding pocket (inhibitor i15 - flexible residues Asn25, Glu48, Glu51, Glu76 , Tyr191 ; inhibitor- i17 : Asn25, Glu51 , Tyr 191; inhibitor- i18 : Glu48, Glu51, Glu76 and Tyr191).

For NNH (Nonspecific Nucleoside Hydrolase) six inhibitors (inhibitor no. i19 to i24) were chosen for docking. Complexes of three of these inhibitors (i22, i23, i24) with a homologous protein from *T. vivax* were available in the PDB (2FF2, 3EPW and 3EPX respectively), whereas the uncomplexed individual structure of NNH from *L. major* was found in 1EZR. The α|β enzyme from *L. major* is a homotetramer with an indispensable calcium ion in its active site [63]. Coordinates for the inhibitors i19, i20 were built starting from the pyrimidine group in the structure of immucilin – H present in 2FF2 and inhibitor i21from the ligand (i23) present in 3EPW. Docking of these inhibitors in the active site of the protein (including the Ca2+ ion), exhibited CHEMPLP scores and rmsd’s (with respect to their original placement) ranging from 50 – 60 and 1.2 – 1.6 Å, respectively. Flexibility was allowed for residues Phe167 and His240 in the enzyme active site during the docking process for all ligands associated with this protein. Interactions with residues Asp 15, Asp 14 (with the exception Inhibitor – i22), Thr 126, Met 152 (except Inhibitor – i20) Asn 160, Glu 166, Phe 167, Asn 168, His 240 , Asp 241 and the calcium ion were common to all the ligands. Contacts with Leu 191 were found only for Inhibitors – i19 and i22 (Additional file 4).

### Group C

Due to the lack of available prototypes or templates in terms of actual crystal structures depicting the position of the ligands in their binding sites, the confidence level associated with the docked complexes in Group C is necessarily low and thus the discussion of these complexes will be fairly brief. The crystal structure trypanothione sythetase (TS) from *L. major* (2VOB) has three putative binding sites for ATP, spermidine and glutathione (GSH). Inhibitor-7 (1-[3-(3-fluorophenyl) indazol-1-yl]-3, 3-dimethylbutan-2-one) binds with uncompetitive inhibition for both the putative ATP and GSH binding sites whereas exhibits competitive inhibition for the site associated with spermidine. Thus, the inhibitor (i25) was placed at the centroid of this site constituted by residues Arg 613, Arg 328, Ser 351, Glu 355, Phe 249 and Glu 407. As mentioned previously, inhibitor – i25 was constructed by *ab initio* fourth atom fixing techniques coupled to energy minimization. Several iterations with flexible ligand and rigid side chains of the active site led to a final CHEMPLP score of 47.32. Introduction of side chain flexibility did not appear to improve final docking poses. Likewise, inhibitor – i26 (ebselen) was positioned in the putative UDP binding pocket of UDP-glc-4′-epimerase (GalE) of *L. major* based on the centroid of residues R335, R268, N202 and H221. GalE from *L. major* was modeled based on the homologous enzyme from *T. brucei (*~58% sequence identity : 1GY8). Coordinates of ebselen were built by the same methods mentioned above and the final docked position had a CHEMPLP score of 36.96. The crystal structure of glyoxalase-I (GLO I) from *L. major* was obtained from 2C21 [44] and S-4-bromobenzylglutathionylspermidine (inhibitor-i27) was docked into the putative active site of the enzyme constituted by residues : A chain - His8, Arg12, Arg33, Trp35, Val37, Glu52, Glu59, Asn63 and B chain - His77, Asp100, Tyr101, Phe107, Met108, Tyr118, Glu120, Met127 and Lys130. The CHEMPLP docking score of 79.43 was obtained for this docking. For all docking runs in case of i27 both the energy minimized ligand and the residues composing the protein active site were held rigid, as additional trials with either flexible ligands and/or side chains led to significant shifts in their position away from the putative binding sites. As mentioned previously the confidence level is relatively low for these complexes.

### Pharmacophore design and screening of zinc database

34 structure based pharmacophores were derived from their corresponding ligand bound three dimensional structures using LigandScout version 3.12 (build 20130912). Those pharmacophores whose area under the ROC curve (See Methods), were less than 0.75 whilst validation, were filtered out (i8, i11, i17 and i20). In addition, pharmacophores with either too few (i2, i20, i26: 3 features) or too many features (i14:13 features, i16:16, i27:12) were removed, leaving a total of 23 pharmacophores for subsequent calculations (Table 4). These pharmacophores were used to search the ZINC database using ZINCPharmer (html search engine) with parameters: ‘Max Hits per Conf’ = 1, ‘Max Hits per Mol’ = 1, ‘Max Total Hits’ = 20 and ‘Max RMSD’ = 0.5, 0.75, 1. The Max RMSD was gradually increased only if no hits were recorded in the initial cutoffs. The topmost hit of every pharmacophore with the least RMSD, along with hits which were similar to approved drugs (generally greater than 90%) are shown in Table 4 and all the hits are given in Additional file 5.A total number of 344 hits were recorded from the ZINC database which were then used to search the Drug Bank (http://www.drugbank.ca) with a cutoff in similarity score set to 70%, so as to identify similar molecules actually in use as pharmaceutical products. From the 344 compounds distributed over 23 pharmacophores, 9 exhibited similarities to drugs under investigation, 319 showed similarities to experimental drugs (known to bind to specific proteins in mammals, bacteria, viruses, fungi, or parasites) and 16 were similar to approved drugs (in at least one country). Of these 344 hits, 40 were from complexes in Group A, 304 from Group B and none from Group C.

**Table 4:** **Results from searching the ZINC Database using structure based pharmacophores**

Inhibitor no.	No. of pharmaco-phoric features	No. of Omitted features	Area Under the ROC Curve (AUC)	SMILES of topmost hit	ZINCID
i1	9	2	0.97	C/C2 = N/C = 1/N = C(/N)N = C(N)C = 1/N = C2/C = 3C = CC([Cl]) = C([Cl])C = 3	ZINC34515729
				**C#CC[C@@](CC = 2C = NC1 = NC(N) = NC(N) = C1N = 2)(C = 3C = CC(=CC = 3)C([O-]) = O)C([O-]) = O**	**ZINC22012802**
				**C#CC[C@](CC = 2C = NC1 = NC(N) = NC(N) = C1N = 2)(C = 3C = CC(=CC = 3)C([O-]) = O)C([O-]) = O**	**ZINC22012807**
				**CC = 1C = CC = C(C = 1)C2 = NC = 3C(/N = C2/N) = NC(N) = NC = 3 N**	**ZINC01566881**
				**C#CC[C@]([H])(CC = 2C = NC1 = NC(N) = NC(N) = C1N = 2)C = 3C = CC(=CC = 3)C([O-]) = O**	**ZINC22012811**
				**C#CC[C@@]([H])(CC = 2C = NC1 = NC(N) = NC(N) = C1N = 2)C = 3C = CC(=CC = 3)C([O-]) = O**	**ZINC22012815**
i3	8	3	0.88	CC = 1C = CC = CC = 1NC(=O)C = 3[S]C = 2/N = C(/N)C(C#N) = CC = 2C = 3 N	ZINC18240380
				**C#CC[C@@]([H])(CC = 2C = NC1 = NC(N) = NC(N) = C1N = 2)C = 3C = CC(=CC = 3)C([O-]) = O**	**ZINC22012815**
i4	6	1	0.97	CN(C)C = [N+]C(=[S])NC = 1C = CC([F]) = CC = 1	ZINC03028809
i5	7	2	0.97	CC = 1/C = C(/C)N = C(N = 1)N/C(=N\C(=[S])NC = 2/C = C(/OC)C([Cl]) = CC = 2OC)N3CCC[C@@](C)([H])C3	ZINC14160212
i6	6	1	0.97	CN(C)C = 1[N+] = CC = CC = 1CNC(N[C@@]2([H])CC[C@@]([H])(C2)[S]C) = [N+]C	ZINC72776752
i7	5	1	0.9	COC = 1C = CC(=CC = 1NC(=[S])NC = 2C = CC([F]) = CC = 2)[N+]([O-]) = O	ZINC00493353
				**C/C1 = C/C = NC = 2C1 = CC(=CC = 2 N[C@@](C)([H])CCC[N+]CCC)OC**	**ZINC01600860**
i9	6	1	0.97	CN(C)C = [N+]C(=[S])NC = 1C = CC([F]) = CC = 1	ZINC03028809
iTb4	6	1	0.97	CN(C)C = [N+]C(=[S])NC = 1C = CC([F]) = CC = 1	ZINC03028809
iTb5	7	2	0.99	CC = 1/C = C(/C)N = C(N = 1)N/C(=N\C(=[S])NC = 2/C = C(/OC)C([Cl]) = CC = 2OC)N3CCC[C@@](C)([H])C3	ZINC14160212
iTb6	7	2	0.98	CC = 1/C = C(/C)N = C(N = 1)N/C(=N/C(=[S])NC = 2C = CC([F]) = CC = 2)NCC = 3C = CC([F]) = CC = 3	ZINC14156881
iTb7	5	1	0.9	C/C1 = C(/C(=NN1C[C@@]2([H])CCC[N+]2C)C([F])([F])[F])C(=O)NCCC[N+](C)C	ZINC49362845
				**C/C1 = C/C = NC = 2C1 = CC(=CC = 2 N[C@@](C)([H])CCC[N+]CCC)OC**	**ZINC01600860**
iTb8	8	3	0.99	CC = 1/C = C(/C)N = C(N = 1)N/C(=N\C(=[S])NC = 2/C = C(/OC)C([Cl]) = CC = 2OC)N3CCC[C@](C)([H])C3	ZINC14160209
i10	7	2	0.91	C/C2 = C/C1 = N/N = C(/[S]CCCCCO)N1C(C) = N2	ZINC72058109
i12	7	3	0.97	CC(=O)NC = 4C = 1/C = C(/[F])C = CC = 1N3C[C@](C)(C(=O)N[C@@]2([H])CCCC2)N(CC[N+](CC)CC)C(=O)C3 = 4	ZINC21866480
i13	4	1	0.99	CC = 1C = CC(=CC = 1)C[N+]2CCC([H])(CC2)CC(N) = O	ZINC40540751
i15	5	2	0.78	C[C@@]1([H])CN(C[C@@](C)([H])O1)[C@]([H])(C(=O)NC = 2C = CC(=CC = 2)NC(=O)COC)C(C)(C)[H]	ZINC58203407
i18	4	1	0.76	C[C@@]1([H])C[C@]([H])(CC)N(C1)C(=O)CC[C@@]2([H])NC(=O)NC2 = O	ZINC73336547
				**CC = 1C = C/C = C(/C)C = 1NC(=O)CNC = 2C = CC = C(C = 2)NC(C) = O**	**ZINC29396021**
i19	6	2	0.93	C[C@@]34CC[C@@]1([H])[C@@]([H])(CC[C@@]2(O)C[C@](O)([H])CC[C@]12/C = [N+]/[C@@]([H])(CC)CO)[C@@]3(O)CC[C@]4([H])C5 = CC(=O)OC5	ZINC09167567
				**C[C@]3([H])O[C@]([H])(O[C@@]1([H])[C@@](O)([H])[C@@](O)([H])[C@]([H])(O[C@]1([H])CO)O[C@@]2([H])[C@@](O)([H])[C@@](O)([H])[C@@](O)([H])O[C@]2([H])CO)[C@](O)([H])[C@](O)([H])[C@]3([H])[N+](C)[C@]4([H])C = C(CO)[C@@](O)([H])[C@@](O)([H])[C@@]4(O)[H]**	**ZINC77302460**
i21	6	1	0.88	O[C@@]1([H])CO[C@@]([H])([C@]1(O)[H])[C@@](O)([H])CO	ZINC05157080
				**C[C@](O)([H])[C@](O)([H])[C@@](O)([H])[C@@](O)([H])CO**	**ZINC03872643**
				**CC(=O)C(=O)[C@@](O)([H])[C@](O)([H])[C@@](O)([H])[C@@](O)([H])CO**	**ZINC64219378**
				**CN/C2 = N/C1 = C(/N = C(/N)NC1 = O)N2[C@]3([H])O[C@]([H])(CO)[C@](O)([H])[C@]3(O)[H]**	**ZINC13361972**
i22	6	2	0.75	OCC(CO)(CO)[N+]C[C@@](O)([H])CN2C1 = C/C = C(/[Br])C = C1C = 3/C = C(/[Br])C = CC2 = 3	ZINC10384387
				**[N+][C@]2([H])C[C@@]([N+])([H])[C@]([H])(O[C@@]1([H])O[C@]([H])(C[N+])[C@](O)([H])[C@@](O)([H])[C@]1(O)[H])[C@@](O)([H])[C@@]2([H])O[C@@]3([H])O[C@]([H])(CO)[C@](O)([H])[C@@]([N+])([H])[C@]3(O)[H]**	**ZINC08214767**
				**C[C@]3(O)CO[C@@]([H])(O[C@]2([H])[C@@]([N+])([H])C[C@]([N+])([H])[C@]([H])(O[C@@]1([H])CC(=CC[C@]1([N+])[H])C[N+])[C@]2(O)[H])[C@@](O)([H])[C@@]3([H])[N+]C**	**ZINC70672630**
				**[N+][C@@]2([H])[C@](O)([H])[C@]([H])(O[C@]1([H])O[C@@]([H])(CO)[C@](O)([H])[C@](O)([H])[C@]1([N+])[H])[C@@]([H])(CO)O[C@]2(O)[H]**	**ZINC43758958**
i23	7	2	0.87	OC = 1C = C/C(=C(/O)C = 1)C2 = NN/C(=C2/C4 = N/C = 3C = CC = CC = 3[S]4)C([F])([F])[F]	ZINC04126006
				**[O-]C(=O)C[C@](O)([H])C[C@](O)([H])C = CC = 2C(=C1C = CC = CC1 = NC = 2[C@]3([H])CC3)C = 4C = CC([F]) = CC = 4**	**ZINC11616582**
i24	7	2	0.87	CC = 1C(O) = CC = C(C = 1O)C2 = NN = C[C@@]2([H])C4 = NC = 3C = CC = CC = 3[S]4	ZINC18188334
				**CN2C = 1/N = C(/NCCO)N(CCO)C = 1C(=O)NC2 = O**	**ZINC01876281**
i25	6	2	0.8	**CC = 1C = CC(=CC = 1)CN2C(=O)C(=CNC2 = O)CC([O-]) = O**	**ZINC20156415**

The topmost hits from the ZINC database utilizing the pharmacophores from the protein – inhibitor complexes. For each pharmacophore the area under the ROC curve and the number of omitted pharmacophoric features while validation have been given in columns 3,4. Also included are hits (given in bold) which have features very similar to approved drugs. The ZINC Id of the hit is given in the last column.

The structure based pharmacophore derived from methotrexate (i1) bound to pteridine reductase returned 20 small molecule compounds (Additional file 5) from the ZINC database, with the pteridine ring being the principal pharmacophoric feature. Most of these compounds from ZINC were variable chemical substitutions around the pteridine ring. Inhibitor i2, i10 and i11 (all complexed with pteridine reductase) failed to give any hit whereas the pharmacophore corresponding to i3 – pteridine reductase, again returned 20 compounds. Two approved drugs pralatrexate and triamterene were found to be similar to hits from pharmacophores involving i1 and i3 (Table 5). Pharmacophores from inhibitors i4,i5,i6,i7,i8,i9 complexed with trypanothione reductase found 20,4,20,20,20 compounds from the ZINC database respectively. For most of these compounds the phenyl ring and the terminal carboxyl (for example in i4 - methyl [(4S) - 6 - bromo - 2 - methyl - 4 - phenylquinazolin - 3(4H)-YL] acetate) appeared to be crucial pharmacophoric features. The approved drug primaquine was found to be similar to the compound ZINC01600860 corresponding to i7. Inhibitors i12 (2 hits), i13 (20 hits) and i14( 0 hit) complexed with parasitic phosphofructokinase failed to find any approved drug from Drugbank, whereas lidocaine and tocainide were found to be similar to ZINC29396021 (i18). For inhibitors i15 (6 hits), i16 (0),i17(0) and i18(20) complexed with deoxy uridine triphosphatase nucleotide hydrolase, the principal pharamacophoric feature(s) responsible for the hits appeared to be the uridyl moiety coupled to the pentose sugar ring. Especially, fruitful were pharmacophores due to complexes with nonspecific nucleoside hydrolase as they yielded acarbose (i19 – 5 hits); mannitol, calcium gluceptate, nelarbine, didanosine, vidarbine (i21 – 20 hits); kanamycin, tobramycin, neomycin, framycetin, paromomycin, gentamicin, glucosamine, netilimicin (i22 – 20 hits); pitavastatin (i23 – 20 hits) and diphylline (i24 – 20 hits). In this case the essential pharmacophoric recognition features were the pyrimidine ring coupled to a pentose sugar. Of the remaining pharmacophores from i25 (20 hits), i26(0),i27(0) complexed with trypanothione synthetase no drug could be recovered from DrugBank. The information with regard to the list of approved drugs has been summarized in Table 5.

**Table 5:** **Approved drugs similar to hits in the ZINC database**

Protein	Inhibitor	ZINC code	Approved drug (drug bank code)	Similarity score
Pteridine	i1	ZINC34515729	Triamterene (DB00384)	0.78
Reductase 1	i1	ZINC01566881	“	0.96
	i1	ZINC22012802	Pralatrexate (DB06813)	0.73
	i1	ZINC22012807	“	0.73
	i1	ZINC22012811	“	0.75
	i1,i3	ZINC22012815	“	0.75
Trypanothione Reductase	i7	ZINC01600860	Primaquine (DB01087)	0.84
Deoxyuridine	i18	ZINC29396021	Lidocaine (DB00281)	0.80
Triphosphatase Nucleotido hydrolase		ZINC29396021	Tocainide (DB01056)	0.72
Nonspecific	i19	ZINC77302460	Acarbose (DB00284)	0.98
Nucleoside	i21	ZINC03872643	Mannitol (DB00742)	1.0
Hydrolase	i21	ZINC64219378	CalciumGluceptate (DB00326)	0.70
	i21	ZINC13361972	Nelarabine (DB01280)	0.76
	i21	ZINC13361972	Didanosine (DB00900)	0.76
	i21	ZINC13361972	Vidarabine (DB00194)	0.76
	i22	ZINC08214767	Kanamycin (DB01172)	1.0
	i22	ZINC43758958	“	0.85
	i22	ZINC08214767	Tobramycin (DB00684)	0.96
	i22	ZINC43758958	“	0.88
	i22	ZINC08214767	Neomycin (Db00094)	0.94
	i22	ZINC43758958	“	0.88
	i22	ZINC08214767	Framycetin (DB00452)	0.94
	i22	ZINC43758958	“	0.88
	i22	ZINC08214767	Paromomycin (DB01421)	0.94
	i22	ZINC43758958	“	0.88
	i22	ZINC08214767	Gentamicin (DB00798)	0.76
	i22	ZINC70672630	“	0.77
	i22	ZINC08214767	Glucosamine(DN01296)	0.74
	i22	ZINC43758958	“	0.88
	i22	ZINC70672630	Netilmicin (DB00955)	0.71
	i23	ZINC11616582	Pitavastatin (DB08860)	1.0
	i24	ZINC01876281	Dyphylline (DB00651)	0.71

Approved drugs with similarity score greater than 0.70 to specific ZINC hits. Information of the protein – inhibitor complex corresponding to the pharmacophore is also given.

Interestingly, the search for approved drugs similar to ZINC compounds led to paromomycin (with a similarity score of 0.944 with respect to immucilin H in complex with nonspecific nucleoside hydrolase, i22)spontaneously appearing in the list, a drug having passed all clinical trials and is now currently being prescribed for visceral leishmaniasis. Paromomycin has also been successfully used in topical creams for the treatment ulcerative cutaneous leishmaniasis [64]. The inclusion of paromomycin provides some confidence that some of the listed drugs (in Table 5) could possibly exhibit some measure of antileishmanial activity. Likewise framycetin, neomycin, gentamicin, netilimicin and tobramycin all belong to the same class of aminoglycoside antibiotics generally known to inhibit protein synthesis. Framycetin and neomycin have found extensive use in topical ointments and creams. Didanosine and vidarbine are antiviral drugs the former being a nucleoside analogue of guanosine with hypoxanthine attached to the sugar ring and the latter an analogue of adenosine, in this case D – arabinose replacing D-ribose. Nelarabine on the other hand is a purine nucleoside analogue currently being applied in the chemotherapy of T-cell acute lymphoblastic leukemia. Other drugs include lidocaine (and its analog tocainide) an amino amide type local anesthetic , primaquine a member of the 8 – aminoquinoline group of drugs used in the treatment of malaria/ pneumocystis pneumonia, pralatrexate an anti-folate for anti-cancer therapy and triamterene a diuretic drug for hypertension. Notably, pteridine reductase, trypanothione reductase, deoxyuridine triphosphatase have been found to be essential for survival and nonspecific nucleoside hydrolase plays a central role in the purine salvage pathway. Currently, our aim is to experimentally test the anti – leishmanial character of these compounds/approved drugs.

## Conclusions

The work reported in this paper demonstrates the series of computational steps beginning with the comparison of genomes, prioritization of prospective drug targets, culling or assembly of inhibitor – target complexes through template based model building and docking, generation of pharmacophores and their subsequent use for searching small molecule databases (such ZINC/Drug Bank), to rationally assemble a set of lead compounds for experimentally testing as potential antileishmanials. The natural appearance of paromomycin, a drug currently being employed against visceral leishmaniais, in the list of lead compounds lends some confidence to the adoption of such scaffold – hopping techniques in order to generate a library of prospective antileishmanials. The next stage of the work will involve experimental validation of these leads.

## Authors’ information

Barnali Waugh and Ambarnil Ghosh are first authors.

## Electronic supplementary material

Additional file 1: **List of prioritized proteins.** (XLSX 14 KB)

Additional file 2: **List of hypothetical proteins.** (XLSX 12 KB)

Additional file 3: **Targeted proteins and their inhibitors.** (XLSX 16 KB)

Additional file 4: **Targeted protein – inhibitor contacts.** (XLSX 2 MB)

Additional file 5: **Hits from the zinc database.** (XLSX 30 KB)
